# Poly[bis­(*N*,*N*-dimethyl­formamide-κ*O*)(μ_4_-naphthalene-1,5-disulfonato)magnesium(II)]

**DOI:** 10.1107/S1600536810017952

**Published:** 2010-05-22

**Authors:** Lauren A. Borkowski, Debasis Banerjee, John B. Parise

**Affiliations:** a Stony Brook University, Mineral Physics Institute, 255 ESS Building, Stony Brook, NY 11794, USA; b Department of Chemistry, Stony Brook University, 255 ESS Building, Stony Brook, NY 11794-2100, USA; c Department of Geosciences, Department of Chemistry, Stony Brook University, 255 ESS Building, Stony Brook, NY 11794-2100, USA

## Abstract

The structure of the title compound, [Mg(C_10_H_6_O_6_S_2_)(C_3_H_7_NO)_2_]_*n*_, consists of MgO_6_ octa­hedra (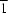 symmetry) connected to naphthalene-1,5-disulfonate ligands (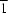 symmetry) in the equatoral plane, forming a two-dimensional network propagating parallel to (010). The coordination sphere of the Mg atom is completed by the O atoms of two *N*,*N*-dimethyl­formamide (DMF) mol­ecules in the axial positions. The title compound represents the first time the naphthalene-1,5-disulfonate anion is bound directly to a Mg^2+^ atom. Disorder over two positions was found in the DMF mol­ecule in a 0.518 (8):0.482 (8) ratio.

## Related literature

For background to metal-organic coordination polymers (CPs) and frameworks (MOFs), see: Cheetham *et al.* (2006 ▶); Kitagawa *et al.* (2004 ▶); Rosseinsky (2004 ▶); Rowsell & Yaghi (2004 ▶). For structures in which Mg^2+^ is not directly linked to naphthalene­disulfonate ligands but is surrounded by water mol­ecules, see: Cody & Hazel (1977 ▶); Morris *et al.* (2003 ▶); Shakeri & Haussühl (1992 ▶).
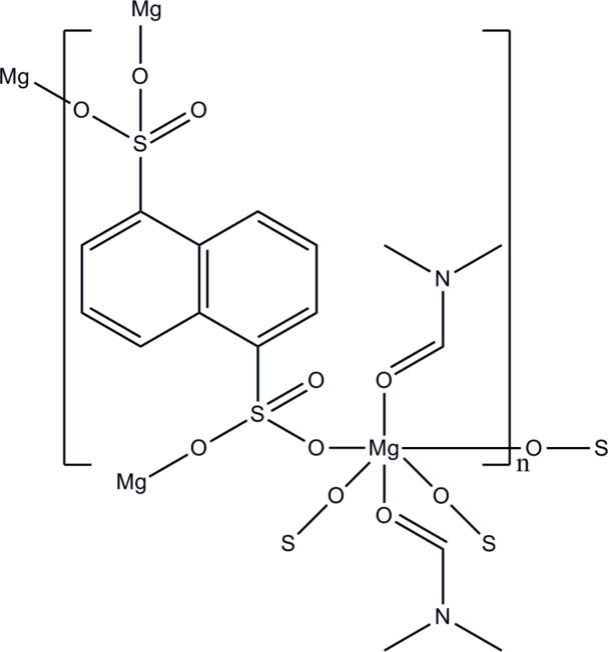


## Experimental

### 

#### Crystal data

[Mg(C_10_H_6_O_6_S_2_)(C_3_H_7_NO)_2_]
*M* *_r_* = 456.79
Triclinic, 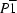

*a* = 5.1328 (2) Å
*b* = 9.3890 (4) Å
*c* = 10.4029 (4) Å
α = 85.158 (1)°
β = 75.638 (1)°
γ = 79.501 (1)°
*V* = 477.13 (3) Å^3^
*Z* = 1
Synchrotron radiation
λ = 0.41328 Å
μ = 0.08 mm^−1^
*T* = 100 K
0.09 × 0.03 × 0.01 mm

#### Data collection

Bruker APEXII CCD diffractometer
Absorption correction: multi-scan (*SADABS*; Bruker, 2008 ▶) *T*_min_ = 0.993, *T*_max_ = 0.999
8811 measured reflections
1961 independent reflections
1721 reflections with *I* > 2σ(*I*)
*R*_int_ = 0.054

#### Refinement

*R*[*F* ^2^ > 2σ(*F* ^2^)] = 0.032
*wR*(*F* ^2^) = 0.087
*S* = 1.04
1961 reflections
221 parameters
6 restraints
Only H-atom coordinates refined
Δρ_max_ = 0.61 e Å^−3^
Δρ_min_ = −0.51 e Å^−3^

### 

Data collection: *APEX2* (Bruker, 2007 ▶); cell refinement: *SAINT-Plus* (Bruker, 2007 ▶); data reduction: *SAINT-Plus*; program(s) used to solve structure: *SIR2004* (Burla *et al.*, 2005 ▶); program(s) used to refine structure: *SHELXL97* (Sheldrick, 2008 ▶); molecular graphics: *CrystalMaker* (CrystalMaker, 2009 ▶); software used to prepare material for publication: *enCIFer* (Allen *et al.*, 2004 ▶).

## Supplementary Material

Crystal structure: contains datablocks global, I. DOI: 10.1107/S1600536810017952/wm2328sup1.cif

Structure factors: contains datablocks I. DOI: 10.1107/S1600536810017952/wm2328Isup2.hkl

Additional supplementary materials:  crystallographic information; 3D view; checkCIF report

## Figures and Tables

**Table 1:** Selected bond lengths (Å)

Mg1—O1	2.0193 (11)
Mg1—O2^i^	2.0429 (11)
Mg1—O4	2.1562 (11)

Symmetry code: (i) 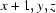.

**Fig. 1.**
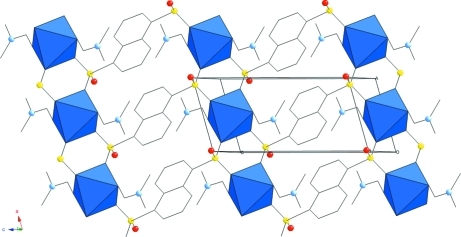
Polyhedral representation of a layer of the title compound. The DMF disorder and the H atoms were omitted for clarity. Color codes: blue octahedra Mg, red spheres O, light blue spheres N, and black lines C.

## supplementary crystallographic information

### Comment

Interest in metal organic coordination polymers (CPs) and frameworks (MOFs) arises due to the potential applications including size/shape catalysis and gas storage/separation (Cheetham *et al.*, 2006; Kitagawa *et al.*, 2004; Rosseinsky, 2004; Rowsell & Yaghi, 2004). This is owing to the fact that these materials have similar properties to zeolites with the added possiblity of structural design. Three factors need to be considered during the sythesis of CPs and MOFs; the coordination sphere of the metal center, the size, shape and functionality of the organic linker and the synthetic conditions.

Previous studies have looked at the interaction between a variety of metal atoms and 1,5-naphthalenedisulfonic acid. A search of the literature results in examples of direct bonding between this ligand and every alkali and alkali earth metal except Mg^2+^. Due to perodic trends we suspected that Mg^2+^ and naphthalene-1,5-disulfonate should have a direct bonding interaction, which indicates a change in synthetic conditions compared to the previous studies was needed. The previous efforts used water as the solvent resulting in structures where the Mg^2+^ atoms are bound solely to water molecules and interacted with 1,5-naphthalenedisulfonic acid through hydrogen bonds (Cody & Hazel, 1977; Morris *et al.*, 2003; Shakeri & Haussühl, 1992). In our study we used *N*,*N*-dimethylformamide (DMF) as the solvent with the thought that DMF is a large solvent molecule and is unlikely to complete for the coordination sphere of the Mg^2+^ atom.

The title compound consists of MgO_6_ octahedra linked by 1,5- naphthalenedisulfonate ligands to form a two-dimensional network parallel to (010). The Mg^2+^ atom is bound to four O atoms from four separate naphthalene-1,5-disulfonate ligands in the equitorial plane and two O atoms from two symmetry equivalent *N*,*N*-dimethylformamide molecules in the axial positions (Figure 1). The average equitorial Mg—O bond length is 2.031 Å whereas the axial bond length is slightly longer at 2.156 Å (Table 1). The individual MgO_6_ octahedra are connected by the naphthalene-1,5-disulfonate linkers in a bridging bidentate fashion to form chains along the [100] direction, which are further connected by the linker molecules to form an overall 2-dimensional structure (Figure 2).

### Experimental

All of the starting materials are available commercially and were used without any further purification. Magnesium nitrate hexahydrate (Mg(NO_3_)_2_^.^6H_2_O, 1.3 grams), 1,5-naphthalenedisulfonic acid tetrahydrate (C_10_H_8_S_2_O_6_ 4H_2_O, 1.87 grams) and ammonium fluoride (NH_4_F, 0.021 grams) were dissolved in 15 grams of *N*,*N*-dimethylformamide (DMF). The solution was prepared in a 23 mL Teflon lined Parr bomb and stirred for three hours to achieve homogeneity. The resulting solution was heated statically at 373 K for three days. Colorless needle shaped crystals were recovered and were washed with ethanol.

### Refinement

Disorder was found in the bound *N*,*N*-dimethylformamide molecules with the major component occupied 51.8 (8)% and the minor 48.2 (8)%, of which only the O atom does not have a second position. The disordered atoms were refined anisotropically without constraints. All of the hydrogen atoms were located in the Fourier difference map and their positions were allowed to refine independent of the C atoms with U_iso_(H)=1.5U_eq_(C) for the methyl groups and U_iso_(H)=1.2U_eq_ (C) for all others. The highest peak (~0.6 e^-^ A^-3^) can be found within 0.89 Å of C2 and the deepest hole (~-0.5 e^-^ A^-3^) within 0.73 Å of S1. The near equal values of the highest peak and deepest hole indicate that there is not any remaining unmodelled electron density. The crystal remained stable throughout the low temperature data collection.

### Crystal data

**Table:**

[Mg(C_10_H_6_O_6_S_2_)(C_3_H_7_NO)_2_]	*Z* = 1
*M**_r_* = 456.79	*F*(000) = 238
Triclinic, *P*1	*D*_x_ = 1.590 Mg m^−^^3^
Hall symbol: -P 1	Synchrotron radiation, λ = 0.41328 Å
*a* = 5.1328 (2) Å	Cell parameters from 4827 reflections
*b* = 9.3890 (4) Å	θ = 2.4–17.1°
*c* = 10.4029 (4) Å	µ = 0.08 mm^−^^1^
α = 85.158 (1)°	*T* = 100 K
β = 75.638 (1)°	Needle, colorless
γ = 79.501 (1)°	0.09 × 0.03 × 0.01 mm
*V* = 477.13 (3) Å^3^	

### Data collection

**Table:**

Bruker APEXII CCD diffractometer	1961 independent reflections
Radiation source: APS Sector 15	1721 reflections with *I* > 2σ(*I*)
graphite	*R*_int_ = 0.054
phi scans	θ_max_ = 15.0°, θ_min_ = 1.3°
Absorption correction: multi-scan (*SADABS*; Bruker, 2008)	*h* = −6→6
*T*_min_ = 0.993, *T*_max_ = 0.999	*k* = −11→11
8811 measured reflections	*l* = −12→12

### Refinement

**Table:**

Refinement on *F*^2^	Primary atom site location: structure-invariant direct methods
Least-squares matrix: full	Secondary atom site location: difference Fourier map
*R*[*F*^2^ > 2σ(*F*^2^)] = 0.032	Hydrogen site location: inferred from neighbouring sites
*wR*(*F*^2^) = 0.087	Only H-atom coordinates refined
*S* = 1.04	*w* = 1/[σ^2^(*F*_o_^2^) + (0.0481*P*)^2^ + 0.2091*P*] where *P* = (*F*_o_^2^ + 2*F*_c_^2^)/3
1961 reflections	(Δ/σ)_max_ < 0.001
221 parameters	Δρ_max_ = 0.61 e Å^−^^3^
6 restraints	Δρ_min_ = −0.51 e Å^−^^3^

### Special details

**Table:**

Geometry. All esds (except the esd in the dihedral angle between two l.s. planes) are estimated using the full covariance matrix. The cell esds are taken into account individually in the estimation of esds in distances, angles and torsion angles; correlations between esds in cell parameters are only used when they are defined by crystal symmetry. An approximate (isotropic) treatment of cell esds is used for estimating esds involving l.s. planes.
Refinement. Refinement of *F*^2^ against ALL reflections. The weighted *R*-factor wR and goodness of fit *S* are based on *F*^2^, conventional *R*-factors *R* are based on *F*, with *F* set to zero for negative *F*^2^. The threshold expression of *F*^2^ > σ(*F*^2^) is used only for calculating *R*-factors(gt) etc. and is not relevant to the choice of reflections for refinement. *R*-factors based on *F*^2^ are statistically about twice as large as those based on *F*, and *R*- factors based on ALL data will be even larger.

### Fractional atomic coordinates and isotropic or equivalent isotropic displacement parameters (Å^2^)

**Table:**

	*x*	*y*	*z*	*U*_iso_*/*U*_eq_	Occ. (<1)
S1	0.07457 (7)	0.11525 (4)	0.80723 (4)	0.00723 (15)	
Mg1	0.5000	0.0000	1.0000	0.00731 (19)	
O1	0.2916 (2)	0.11924 (12)	0.87562 (11)	0.0100 (3)	
O2	−0.1359 (2)	0.04071 (13)	0.88296 (11)	0.0117 (3)	
O3	−0.0320 (2)	0.25885 (12)	0.76873 (12)	0.0123 (3)	
O4	0.5528 (2)	−0.18505 (12)	0.88102 (12)	0.0115 (3)	
C1	0.4500 (3)	0.04297 (17)	0.56082 (16)	0.0084 (3)	
C2	0.2241 (3)	0.00625 (17)	0.65774 (15)	0.0088 (3)	
C3	0.1051 (3)	−0.10819 (18)	0.63682 (17)	0.0113 (3)	
H3	−0.048 (4)	−0.127 (2)	0.698 (2)	0.014*	
C4	0.4211 (3)	−0.16097 (18)	0.42018 (17)	0.0103 (3)	
H4	0.474 (4)	−0.216 (2)	0.343 (2)	0.012*	
C5	0.2054 (4)	−0.19167 (19)	0.51598 (17)	0.0135 (4)	
H5	0.112 (4)	−0.269 (2)	0.503 (2)	0.016*	
C6	0.3941 (13)	−0.2695 (7)	0.8776 (6)	0.0152 (12)	0.518 (8)
H6	0.213 (9)	−0.258 (4)	0.935 (4)	0.018*	0.518 (8)
N1	0.4566 (15)	−0.3857 (7)	0.7960 (6)	0.0128 (13)	0.518 (8)
C7	0.2600 (9)	−0.4804 (5)	0.7982 (5)	0.0269 (13)	0.518 (8)
H7A	0.355 (14)	−0.580 (7)	0.827 (7)	0.040*	0.518 (8)
H7B	0.089 (11)	−0.435 (6)	0.866 (5)	0.040*	0.518 (8)
H7C	0.224 (10)	−0.489 (6)	0.713 (6)	0.040*	0.518 (8)
C8	0.7147 (16)	−0.4185 (7)	0.6958 (6)	0.0182 (13)	0.518 (8)
H8A	0.809 (13)	−0.505 (9)	0.686 (7)	0.027*	0.518 (8)
H8B	0.683 (11)	−0.383 (6)	0.620 (5)	0.027*	0.518 (8)
H8C	0.839 (10)	−0.384 (5)	0.721 (5)	0.027*	0.518 (8)
C6'	0.4544 (12)	−0.2955 (6)	0.9149 (6)	0.0098 (11)	0.482 (8)
H6'	0.362 (9)	−0.313 (5)	1.007 (5)	0.012*	0.482 (8)
N1'	0.4704 (16)	−0.4086 (8)	0.8350 (7)	0.0108 (13)	0.482 (8)
C7'	0.3529 (8)	−0.5363 (4)	0.8827 (4)	0.0154 (13)	0.482 (8)
H7A'	0.228 (11)	−0.544 (8)	0.839 (6)	0.023*	0.482 (8)
H7B'	0.500 (10)	−0.625 (5)	0.860 (5)	0.023*	0.482 (8)
H7C'	0.279 (9)	−0.520 (5)	0.977 (5)	0.023*	0.482 (8)
C8'	0.6042 (17)	−0.4110 (8)	0.6929 (6)	0.0157 (14)	0.482 (8)
H8A'	0.751 (13)	−0.511 (9)	0.678 (7)	0.024*	0.482 (8)
H8B'	0.674 (10)	−0.320 (6)	0.674 (5)	0.024*	0.482 (8)
H8C'	0.469 (11)	−0.418 (5)	0.651 (5)	0.024*	0.482 (8)

### Atomic displacement parameters (Å^2^)

**Table:**

	*U*^11^	*U*^22^	*U*^33^	*U*^12^	*U*^13^	*U*^23^
S1	0.0075 (2)	0.0079 (2)	0.0062 (2)	−0.00348 (15)	−0.00026 (15)	0.00097 (15)
Mg1	0.0080 (4)	0.0073 (4)	0.0069 (4)	−0.0044 (3)	−0.0006 (3)	0.0014 (3)
O1	0.0109 (5)	0.0108 (6)	0.0091 (6)	−0.0057 (4)	−0.0027 (4)	0.0039 (5)
O2	0.0097 (5)	0.0156 (6)	0.0103 (6)	−0.0080 (5)	0.0006 (4)	0.0010 (5)
O3	0.0140 (6)	0.0089 (6)	0.0134 (6)	−0.0014 (5)	−0.0038 (5)	0.0028 (5)
O4	0.0138 (6)	0.0098 (6)	0.0116 (6)	−0.0058 (5)	−0.0016 (5)	0.0002 (5)
C1	0.0093 (7)	0.0077 (8)	0.0085 (8)	−0.0017 (6)	−0.0031 (6)	0.0018 (6)
C2	0.0099 (7)	0.0090 (8)	0.0072 (8)	−0.0012 (6)	−0.0022 (6)	0.0009 (6)
C3	0.0116 (8)	0.0126 (8)	0.0098 (8)	−0.0062 (6)	−0.0005 (6)	0.0025 (6)
C4	0.0138 (8)	0.0093 (8)	0.0083 (8)	−0.0051 (6)	−0.0013 (6)	−0.0007 (6)
C5	0.0173 (8)	0.0120 (8)	0.0134 (8)	−0.0104 (7)	−0.0019 (7)	−0.0004 (7)
C6	0.016 (3)	0.020 (3)	0.012 (3)	−0.0077 (19)	−0.0046 (19)	−0.001 (2)
N1	0.020 (2)	0.010 (2)	0.011 (3)	−0.0105 (18)	−0.004 (2)	0.002 (2)
C7	0.032 (2)	0.022 (2)	0.031 (3)	−0.0188 (19)	−0.0045 (19)	−0.0054 (19)
C8	0.020 (3)	0.015 (3)	0.017 (2)	−0.001 (3)	−0.002 (3)	−0.0009 (16)
C6'	0.010 (2)	0.011 (2)	0.010 (3)	−0.0038 (18)	−0.0023 (18)	−0.004 (2)
N1'	0.012 (2)	0.008 (2)	0.010 (3)	−0.0034 (17)	0.000 (2)	0.002 (2)
C7'	0.0156 (19)	0.008 (2)	0.022 (3)	−0.0054 (14)	−0.0012 (16)	−0.0012 (16)
C8'	0.018 (3)	0.016 (2)	0.014 (3)	−0.004 (3)	−0.003 (3)	−0.0018 (16)

### Geometric parameters (Å, °)

**Table:**

S1—O3	1.4234 (12)	C5—H5	0.98 (2)
S1—O2	1.4276 (12)	C6—N1	1.381 (8)
S1—O1	1.4701 (11)	C6—H6	0.97 (4)
S1—C2	1.8560 (16)	N1—C7	1.456 (7)
Mg1—O1	2.0193 (11)	N1—C8	1.469 (9)
Mg1—O1^i^	2.0193 (11)	C7—H7A	1.03 (7)
Mg1—O2^ii^	2.0429 (11)	C7—H7B	1.03 (6)
Mg1—O2^iii^	2.0429 (11)	C7—H7C	0.97 (6)
Mg1—O4^i^	2.1562 (11)	C8—H8A	0.87 (9)
Mg1—O4	2.1562 (11)	C8—H8B	0.87 (5)
O2—Mg1^iv^	2.0429 (11)	C8—H8C	0.87 (5)
O4—C6'	1.223 (6)	C6'—N1'	1.382 (8)
O4—C6	1.244 (6)	C6'—H6'	0.97 (5)
C1—C2	1.409 (2)	N1'—C7'	1.436 (7)
C1—C4^v^	1.440 (2)	N1'—C8'	1.468 (8)
C1—C1^v^	1.488 (3)	C7'—H7A'	0.89 (6)
C2—C3	1.386 (2)	C7'—H7B'	1.02 (5)
C3—C5	1.469 (2)	C7'—H7C'	0.97 (5)
C3—H3	0.92 (2)	C8'—H8A'	1.09 (9)
C4—C5	1.350 (2)	C8'—H8B'	0.98 (5)
C4—C1^v^	1.440 (2)	C8'—H8C'	0.92 (5)
C4—H4	0.95 (2)		
			
O3—S1—O2	111.65 (7)	C3—C5—H5	119.7 (12)
O3—S1—O1	109.59 (7)	O4—C6—N1	124.7 (6)
O2—S1—O1	113.33 (7)	O4—C6—H6	120 (2)
O3—S1—C2	109.78 (7)	N1—C6—H6	115 (2)
O2—S1—C2	104.07 (7)	O4—C6—H6'	94.7 (18)
O1—S1—C2	108.22 (7)	N1—C6—H6'	111.8 (18)
O1—Mg1—O1^i^	180.00 (4)	H6—C6—H6'	61 (3)
O1—Mg1—O2^ii^	91.70 (5)	C6—N1—C7	121.5 (6)
O1^i^—Mg1—O2^ii^	88.30 (5)	C6—N1—C8	123.5 (6)
O1—Mg1—O2^iii^	88.30 (5)	C7—N1—C8	114.9 (6)
O1^i^—Mg1—O2^iii^	91.70 (5)	N1—C7—H7A	103 (4)
O2^ii^—Mg1—O2^iii^	180.0	N1—C7—H7B	104 (3)
O1—Mg1—O4^i^	90.98 (5)	H7A—C7—H7B	115 (5)
O1^i^—Mg1—O4^i^	89.02 (5)	N1—C7—H7C	114 (3)
O2^ii^—Mg1—O4^i^	93.38 (5)	H7A—C7—H7C	108 (5)
O2^iii^—Mg1—O4^i^	86.62 (4)	H7B—C7—H7C	112 (4)
O1—Mg1—O4	89.02 (5)	N1—C8—H8A	123 (5)
O1^i^—Mg1—O4	90.98 (5)	N1—C8—H8B	107 (4)
O2^ii^—Mg1—O4	86.62 (4)	H8A—C8—H8B	110 (6)
O2^iii^—Mg1—O4	93.38 (5)	N1—C8—H8C	108 (3)
O4^i^—Mg1—O4	180.0	H8A—C8—H8C	94 (5)
S1—O1—Mg1	141.82 (7)	H8B—C8—H8C	115 (5)
S1—O2—Mg1^iv^	161.80 (8)	N1'—C6'—H6'	113 (2)
C6'—O4—Mg1	126.6 (3)	C6'—N1'—C7'	123.1 (5)
C6—O4—Mg1	130.9 (3)	C6'—N1'—C8'	124.2 (6)
C2—C1—C4^v^	120.44 (15)	C7'—N1'—C8'	112.7 (6)
C2—C1—C1^v^	118.31 (17)	N1'—C7'—H7A'	109 (5)
C4^v^—C1—C1^v^	121.26 (18)	N1'—C7'—H7B'	109 (3)
C3—C2—C1	119.08 (15)	H7A'—C7'—H7B'	106 (5)
C3—C2—S1	120.03 (12)	N1'—C7'—H7C'	103 (3)
C1—C2—S1	120.80 (12)	H7A'—C7'—H7C'	114 (5)
C2—C3—C5	122.01 (15)	H7B'—C7'—H7C'	116 (4)
C2—C3—H3	117.9 (12)	N1'—C8'—H8A'	107 (4)
C5—C3—H3	119.9 (12)	N1'—C8'—H8B'	104 (3)
C5—C4—C1^v^	118.10 (15)	H8A'—C8'—H8B'	118 (5)
C5—C4—H4	118.1 (12)	N1'—C8'—H8C'	105 (3)
C1^v^—C4—H4	123.7 (12)	H8A'—C8'—H8C'	107 (4)
C4—C5—C3	121.24 (15)	H8B'—C8'—H8C'	115 (4)
C4—C5—H5	119.0 (12)		

Symmetry codes: (i) −*x*+1, −*y*, −*z*+2; (ii) *x*+1, *y*, *z*; (iii) −*x*, −*y*, −*z*+2; (iv) *x*−1, *y*, *z*; (v) −*x*+1, −*y*, −*z*+1.
